# Impact of Air Conditioning Systems on the Outdoor Thermal Environment during Summer in Berlin, Germany

**DOI:** 10.3390/ijerph17134645

**Published:** 2020-06-28

**Authors:** Luxi Jin, Sebastian Schubert, Mohamed Hefny Salim, Christoph Schneider

**Affiliations:** 1 Geography Department, Humboldt-Universität zu Berlin, 10117 Berlin, Germany; sebastian.schubert@geo.hu-berlin.de (S.S); mohamed.salim@geo.hu-berlin.de (M.H.S.); christoph.schneider@geo.hu-berlin.de (C.S.); 2 Faculty of Energy Engineering, Aswan University, Aswan 81528, Egypt

**Keywords:** air conditioning systems, anthropogenic heat, COSMO-CLM, building energy model, urban heat island

## Abstract

This study investigates the effect of anthropogenic heat emissions from air conditioning systems (AC) on air temperature and AC energy consumption in Berlin, Germany. We conduct simulations applying the model system CCLM/DCEP-BEM, a coupled system of the mesoscale climate model COSMO-CLM (CCLM) and the urban Double Canyon Effect Parameterization scheme with a building energy model (DCEP-BEM), for a summer period of 2018. The DCEP-BEM model is designed to explicitly compute the anthropogenic heat emissions from urban buildings and the heat flux transfer between buildings and the atmosphere. We investigate two locations where the AC outdoor units are installed: either on the wall of a building (VerAC) or on the rooftop of a building (HorAC). AC waste heat emissions considerably increase the near-surface air temperature. Compared to a reference scenario without AC systems, the VerAC scenario with a target indoor temperature of 22 ${}^{\circ }C$ results in a temperature increase of up to 0.6 K. The increase is more pronounced during the night and for urban areas. The effect of HorAC on air temperature is overall smaller than in VerAC. With the target indoor temperature of 22 ${}^{\circ }C$, an urban site’s daily average AC energy consumption per floor area of a room is 9.1 $W\;m^{2}$, which is 35% more than that of a suburban site. This energy-saving results from the urban heat island effect and different building parameters between both sits. The maximum AC energy consumption occurs in the afternoon. When the target indoor temperature rises, the AC energy consumption decreases at a rate of about 16% per 2 K change in indoor temperature. The nighttime near-surface temperature in VerAC scenarios shows a declining trend (0.06 K per 2 K change) with increasing target indoor temperature. This feature is not obvious in HorAC scenarios which further confirms that HorAC has a smaller impact on near-surface air temperature.

## 1. Introduction

The United Nations anticipate that 68% of the world’s population will live in cities by 2050, compared to today’s statistics of 55% [1]. Projections show that the expansion of urban areas and urban activities will increase the indoor energy consumption, and hence increase urban air pollution and climate emissions [2]. These changes have an immediate impact on urban residents’ health and well-being. Previous studies report that heat waves and the subsequently increased concentration of air pollutants are a major threat to human health in metropolitan areas [3,4,5,6]. In fact, the anthropogenic heat emissions from residential and commercial buildings contribute to the urban heat island (UHI) phenomenon [7,8,9,10,11], intensify pollutant concentration over urban areas [12] and impact local atmospheric conditions [13].

In summer, air conditioning (AC) load represents the main source of the anthropogenic heat emissions [14]. Since AC energy demand depends on the outdoor air temperature, AC affects the intensity of UHI and vise versa, as confirmed in several studies for many cities [15,16,17,18,19]. Some studies reported that cooling energy demand for AC is increased by urban expansion and increasing UHI [20,21]. The AC energy demand varies among cities. For instance, 0.5 $W\;m^{-2}$–2.7 $W\;m^{-2}$ is calculated by Salamanca et al. [21] for the city of Phoenix with different AC setups. In Asian cities with denser and higher buildings, the AC electricity consumption is often higher. Kikegawa et al. [22] estimated the AC energy consumption during daytime in summer in Tokyo to be 220 $W\;m^{-2}$ and Xu et al. [23] reported a value up to 146 $W\;m^{-2}$ in Beijing. The anthropogenic heat emission from AC affects the thermal stratification of the boundary layer by increasing vertical mixing during the night in urban areas [20]. It also increases the instability of the urban boundary layer in the morning and evening [24]. Although the release of waste heat due to AC is greatest at noon in summer, the effect on air temperatures is more pronounced during the night [20,24,25,26].

Numerical models are often utilized to quantitatively assess the impact of AC systems on urban meteorology. For instance, Kikegawa et al. [27] presented the building energy analysis model (Kikegawa-BEM) which explicitly calculates the cooling load inside the buildings and accounts for the response of the waste heat from the AC systems to the urban atmosphere. This model has been coupled with various meteorological models in many studies. For example, Ohashi et al. [28] coupled BEM with a canopy meteorological model (CM-BEM) and employed it for Tokyo (Japan) and showed that the air temperature was increased by 1 K–2 K due to AC on weekdays within commercial buildings. Kikegawa et al. [22] implemented the coupled CM-BEM model in the mesoscale weather research and forecasting model (WRF) and derived a rate of temperature increase of up to 1 K per 100 $W\;m^{2}$ emitted anthropogenic heat for downtown areas in Japan.

Similarly, Salamanca et al. [29] developed a building energy model integrated in a multi-layer building effect parametrization (BEP-BEM) which is able to dynamically calculate the exchange of energy between the buildings and the outdoor environment as well as the effect of the AC systems. Salamanca et al. [20] and Salamanca et al. [21] applied a coupled system of BEP-BEM and WRF. They report an increase in mean 2 m temperature by up to 1.75 K due to AC in a semi-arid area (Phoenix, United States) in summer. Similarly for Madrid (Spain) in summer, air temperature is increased by AC up to 1.5 K–2 K [25,30]. Another attempt regarding resolving the urban energy mechanism is the town energy budget (TEB) [31] which also calculates heat fluxes from buildings by dynamically solving the energy budget equation. Tremeac et al. [32] and De Munck et al. [26] utilized the TEB model with the mesoscale meteorological model MESO-NH [33] to analyze the influence of the AC management in Paris (France). Results showed that AC systems increase air temperatures from 0.5 K–2 K for Paris in summer depending on the types of AC.

This study focuses on analyzing the interactions between anthropogenic heat emissions from the AC systems and the outdoor thermal environment in Berlin (Germany) during a dry and hot summer period for different scenarios. To this end, we show multiple simulations using the mesoscale non-hydrostatic regional climate model COSMO-CLM (CCLM, COnsortium for Small-scale MOdeling in CLimate Mode) [34] coupled with the multi-layer Double Canyon Effect Parametrization scheme ([DCEP]) [35] integrated with a building energy model ([DCEP-BEM]) [36]. The model system CCLM/DCEP-BEM is described in details in Jin et al. [36].

This paper is structured as follows: Section 2 describes the model and its set-up including the two different kinds of AC systems. Section 3 presents the results of the different CCLM/DCEP-BEM simulations which are discussed in Section 4.

## 2. Model Description and Set-Up

### 2.1. Model System CCLM/DCEP-BEM

The CCLM [34] is the climate mode of the numerical weather forecast model COSMO which originates from the operational weather forecast Local Model (LM) developed by the German Meteorological Service (DWD) [37]. The CCLM has been jointly developed by the European Consortium COSMO and organizations within the Regional Climate Research community CLM-community (http://www.clm-community.eu)). In this sense, the CCLM is a uniform model system for both numerical weather forecasting and regional climate modeling. Simulations with CCLM have time spans up to centuries and spatial grid spacings from 0.25–50 km.

The Double-Canyon Effect Parametrization scheme (DCEP; Schubert et al. [35]) is a multi-layer urban parametrization scheme which computes the momentum, the sensible heat fluxes and the radiation budget of urban surfaces by utilizing incoming radiation and various meteorological variables from a mesoscale climate model. DCEP is used to represent urban areas and describes the urban structure as quasi two-dimensional street canyons consisting of a ground surface and a row of buildings with two walls. When coupled with the CCLM, CCLM/DCEP provides a realistic representation for the typical characteristics of the urban boundary layer [38].

The DCEP has been further developed by integrating a building energy model (BEM) (DCEP-BEM; Jin et al. [36]) which is based on the BEM presented by Salamanca et al. [29]. DCEP-BEM calculates the anthropogenic heat effect of urban buildings and considers the interaction between the building interior and the outdoor atmosphere which is accomplished by the radiation transfer through the windows, the heat diffusion through the building structures or the natural ventilation and the indoor heating or cooling systems. DCEP-BEM has been coupled with CCLM [36] and it is shown that the performance of the coupled system CCLM/DCEP-BEM in representing urban heat island effects is improved compared to CCLM/DCEP.

In the following we only present the implementation of AC systems into DCEP-BEM. Please refer to Jin et al. [36] for a full model description.

### 2.2. Air Conditioning Configurations

DCEP-BEM computes the indoor temperature ($T_{\mathrm{in}}$) as a function of the total indoor sensible heat fluxes which stem from the wall conduction, anthropogenic heat from humans and devices within the building as well as ventilation. $T_{\mathrm{in}}$ can be controlled when enabling the AC systems in DCEP-BEM, i.e., when setting a target indoor temperature and humidity. The sensible and latent heat flux required for maintaining the target environment are provided by the AC systems. The AC energy consumption is subsequently estimated. In addition, AC systems directly reject extra waste heat through outdoor units to the atmosphere and consequently affect outdoor air temperature. The waste heat consists of the heat removed from the interior of an occupied space and the consumed energy of the AC systems themselves.

**Figure 1:**
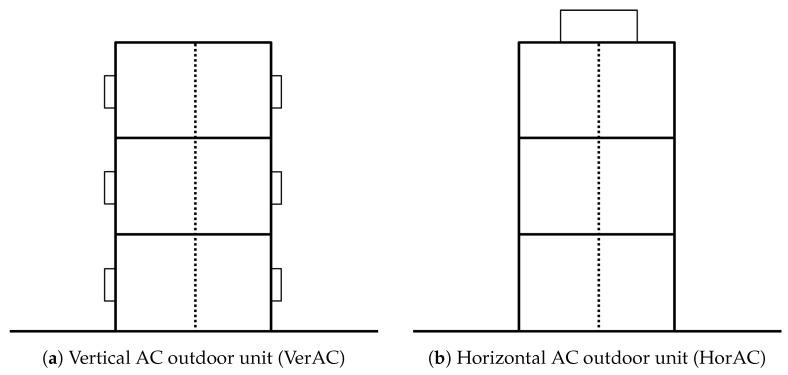
Illustration of the air conditioning (AC) outdoor units in Double Canyon Effect Parameterization scheme with a building energy model (DCEP-BEM). Each floor is divided into two sub-rooms (shown in dashed line). The boxes on the walls and the rooftop denote the AC outdoor units for (**a**) VerAC and (**b**) HorAC configurations, respectively. Each unit in VerAC case is responsible for each sub-room. The unit of the HorAC case ejects the waste heat of the whole building block.

In order to study the impact of the location of AC outdoor units on air temperature, two types of outdoor units are considered in DCEP-BEM. In the VerAC scenario, the units are located on the vertical walls (Figure 1a) with each floor having its own outdoor unit, emitting their waste heat vertically into the atmosphere. In the HorAC scenario, the unit is located on the rooftop of each building (Figure 1b), controlling the indoor environment for the whole building and releasing its total waste heat into the atmosphere from the rooftop. In addition to these realistic settings, an idealized scenario AC_noout (cf. Section 1) is also applied.

### 2.3. Simulation Setup

**Figure 2:**
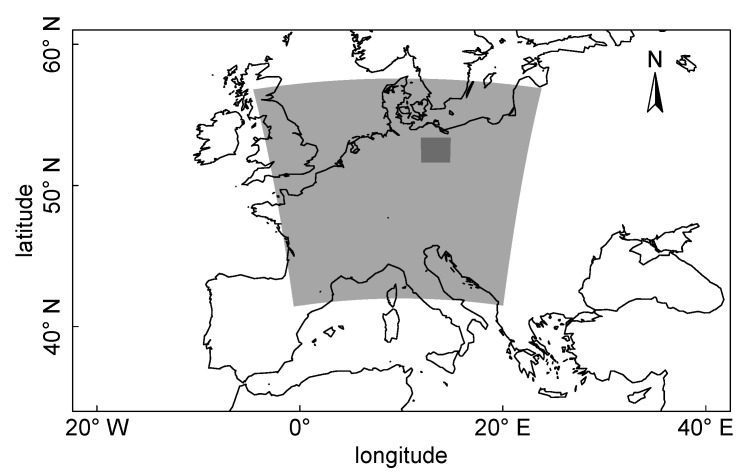
Nested domains of a two-step one-way dynamical downscaling driven by the gloabal reanalysis dataset ERA5 with a spatial resolution of 31 km. The outer domain (central Europe) and the inner study area (city of Berlin, Germany) have grid spacing of 7 km and 1 km, respectively. The map uses a cylindrical equidistant projection.

We apply the version COSMO5.0-CLM9 in this study. The model domain consists of two nested sub-domains with a horizontal grid spacing of 7 km and 1 km (Figure 2). The larger domain (CCLM-7 km) covers central Europe with a domain size of 250 × 250 grid points. The inner domain (CCLM-1 km) covers the area of Berlin with 195 × 195 grid points. The vertical resolution of CCLM-1 km is composed of 52 levels stretching up to 22,000 m, the lowest of which is 5 m above the ground.

The physical parameterizations employed in the CCLM runs are listed in Table 1. The timestep of CCLM-7 km and CCLM-1 km is 40 s and 10 s, respectively. In addition, a multi-layer soil model with a vegetation parametrization is applied in both resolutions. Regarding the microphysics scheme, CCLM-7 km includes cloud ice in representing precipitation formation in water, mixed phase and ice clouds. CCLM-1 km additionally involves graupel phase to the hydrological cycle [39].

The global reanalysis dataset ERA5 [48] provides the initial and lateral boundary conditions for the coarser simulation CCLM-7 km. External parameters (e.g., monthly vegetation and soil parameters) for the preprocessor of the coarser simulation are obtained with the EXTPAR software system WebPEP (https://www.clm-community.eu/) (External Parameter for Numerical Weather Prediction and Climate Application) [49]. The urban scheme DCEP-BEM is applied to the finest resolution run CCLM-1 km. While for CCLM without urban schemes, the above-mentioned external parameters represent the whole grid cell, with DCEP-BEM, a grid cell is divided into a vegetated part with vegetation-related parameters and an urban part without those parameters. To get a realistic mean value for the vegetated part of the finer domain of Berlin, the values from the surroundings of Berlin are adopted. A leaf area index of 3.5 m${}^{2}$ $m^{-2}$, a plant cover fraction of 0.88, a root depth of 1.5 m and a roughness length of 0.13 m are used.

**Figure 3:**
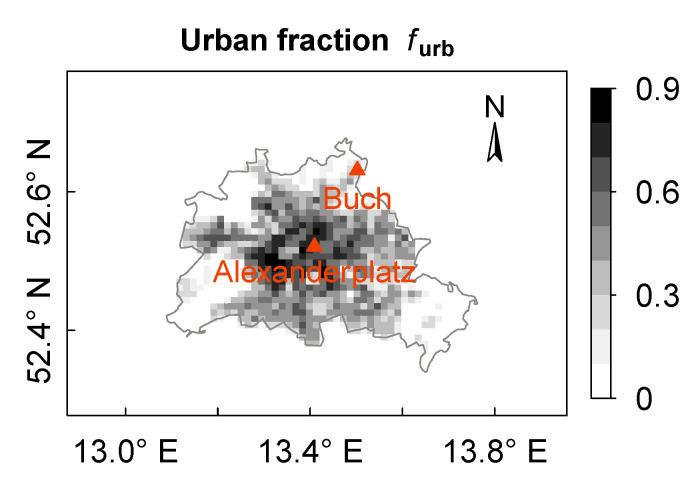
Urban fraction ($f_{\mathrm{urb}}$) of the city area of Berlin. Triangles indicate the observational sites. The city border of Berlin is depicted with the grey line. The map uses a cylindrical equidistant projection. The grid spacing is about 1 km.

The derivation of the urban structure and the canopy parameters (UCPs) of DCEP-BEM for Berlin is based on a dataset with over 460,000 3-D buildings and is explained in Schubert and Grossman-Clarke [50]. In this approach, the urban fraction parameter of a grid cell is equal to the fraction of impervious surfaces in reality (Figure 3). Building width parameters are chosen such that the fraction of buildings in the model corresponds to that in reality. Four street directions (${-45}^{\circ }$, $0^{\circ }$, $45^{\circ }$, $90^{\circ }$ from north) are considered with spatially resolved UCPs. The height of each floor of the buildings is set to 5 m. The emissivity ϵ and thermal diffusivity *k* of the urban surfaces, i.e., roof (R), wall (W) and ground (G), are based on Martilli et al. [51]: $\epsilon_{R}$ = $\epsilon_{W}$ = 0.90, $\epsilon_{G}$ = 0.95, $k_{R}$ = $0.67{\;\times \;10}^{-6}$ m $s^{-2}$ and $k_{G}$ = $0.29{\;\times \;10}^{-6}$ m $s^{-2}$. For consideration of the insulation of the exterior wall in DCEP-BEM, we set $k_{W}$ = $0.67{\;\times \;10}^{-6}$ m $s^{-2}$ for all layers of the wall but the innermost layer ($0.01{\;\times \;10}^{-6}$ m $s^{-2}$). The heat capacity *c* is adopted from Roberts et al. [52]: $c_{R}$ = $1.769{\;\times \;10}^{6}$ J $m^{-1}$ $K^{-3}$, $c_{W}$ = $2.250{\;\times \;10}^{6}$ J $m^{-3}$ $K^{-1}$ and $c_{G}$ = $1.940{\;\times \;10}^{6}$ J $m^{-3}$ $K^{-1}$. The values of albedo (α) follow Roessner et al. [53] and Schubert and Grossman-Clarke [50]: $\alpha_{W}$ = 0.162 and $\alpha_{R}$ = $\alpha_{G}$ = 0.163.

The thickness of the external wall, internal wall, roof of the building and the ground of the street canyon is 0.32 m, 0.20 m, 0.16 m and 0.54 m, respectively. The area fraction of windows in the external wall is set to 0.16. The temperatures of the urban surfaces are initialized with 22.85 ${}^{\circ }C$. The density of persons in the buildings is set to 0.026 person $\;m^{-2}$ [54]. A sensible heat production of individuals of 160 W for the daily activities was suggested by Salamanca and Martilli [55], and about 70 W was proposed for the resting period [56]. Assuming the ratio of activity and resting is 2:1, we obtain a daily average sensible heat production of 130 W per person. The metabolic latent heat production and sensible heat generation due to equipment (except for heating or cooling devices) are set to 22.7 W per person, and 7.4 W $m^{-2}$, respectively, following Salamanca and Martilli [55].

We conduct 12 simulations with CCLM/DCEP-BEM from 20 July 2018 0000UTC to 4 August 2018 2300 UTC. The first 3 days are considered as spin-up period for each simulation. The remaining 13 days (from 23 July 2018 0000 UTC to 4 August 2018 2300 UTC) are denoted as “analysis period”. The 12 simulations consist of: (1) one standard reference run (NoAC), where $T_{\mathrm{in}}$ is not fixed. The NoAC scenario represents the current situation in Germany where AC systems are usually not deployed in residential buildings [57]. (2) One reference run (AC_noout22), where $T_{\mathrm{in}}$ is set to 22 ${}^{\circ }C$ as the target temperature for the AC systems but the heat emissions from AC are not released to the atmosphere (cf. similar studies [20,21,30]). In fact, AC_noout could be interpreted as storing the waste heat in the ground. (3) Five scenarios with different target temperatures (18 ${}^{\circ }C$, 20 ${}^{\circ }C$, 22 ${}^{\circ }C$, 24 ${}^{\circ }C$ and 26 ${}^{\circ }C$), using vertical AC outdoor units (VerAC18, VerAC20, VerAC22, VerAC24, VerAC26) and (4) five scenarios with the same target temperatures as in 3 but applying horizontal AC outdoor unit (HorAC18, HorAC20, HorAC22, HorAC24, HorAC26). The main analysis of the different cases with AC systems is done for a target indoor temperature of 22 ${}^{\circ }C$.

For the scenario NoAC, the natural ventilation rate is set to 0.25, i.e., 25% total volume of the indoor air is exchanged with the outdoor air per hour, considering that dwellers may open the window and vent a room regularly during the day. The natural ventilation is not applicable for the AC enabled scenarios. The NoAC scenario with the present set-up for Berlin was extensively evaluated in Jin et al. [36] against the observations—CCLM/DCEP-BEM shows a good performance for atmospheric radiation, surface energy fluxes and air temperature and is able to capture the urban heat island phenomenon. By comparing with the rural reference site Lindenberg (close to Berlin), the mean-bias error (MBE) and root-mean-square error (RMSE) of the urban island intensity at an urban site (Alexanderplatz) in Berlin during a summer are −0.7 K and 1.8 K, respectively. The values of MBE and RMSE for a suburban site (Buch) are 0.0 K and 2.2 K, respectively.

### 2.4. Geographical and Meteorological Background

The study area is the city of Berlin, the largest city in Germany, which is situated in northeastern Germany between 52.3 ${}^{\circ }$ N to 52.7 ${}^{\circ }$ N and 13 ${}^{\circ }$ E to 13.8 ${}^{\circ }$ E. Human settlement (including buildings and streets) covers 70.5% of the areas in Berlin. Berlin holds around 3.7 million inhabitants (2018) [58]. Berlin has a relatively flat terrain with many lakes and rivers and its climate is denoted as temperate-oceanic (Köppen-Geiger: Cfb) [59].

In order to study different types of urban environments in Berlin, two sites with distinct urban topologies are selected as representative urban and suburban sites (cf. Figure 3). The first site, Alexanderplatz, is located in the densely built-up city center (52.5208 ${}^{\circ }$ N, 13.4094 ${}^{\circ }$ E) surrounded by small scattered vegetation. The mean building height for the grid cell of this site is 25 m. Based on the World Urban Database and Access Portal Tools (WUDAPT) [60,61] and the Local Climate Zones classification (LCZ) [62], the site Alexanderplatz is classified as LCZ2 (compact mid-rise) according to the WUDAPT–LCZ approach [63]. Therefore, the site Alexanderplatz is denoted as an “urban site” in this work. The second site, Buch, is located on the northeastern outskirt of Berlin (52.6309 ${}^{\circ }$ N, 13.5022 ${}^{\circ }$ E), and is classified as LCZ6 (open low-rise) [63]. The district of Buch has dominant low-rise residential buildings (with mean building height of 15 m) and low population density. Hence, the site Buch is considered as a “suburban site”.

**Figure 4:**
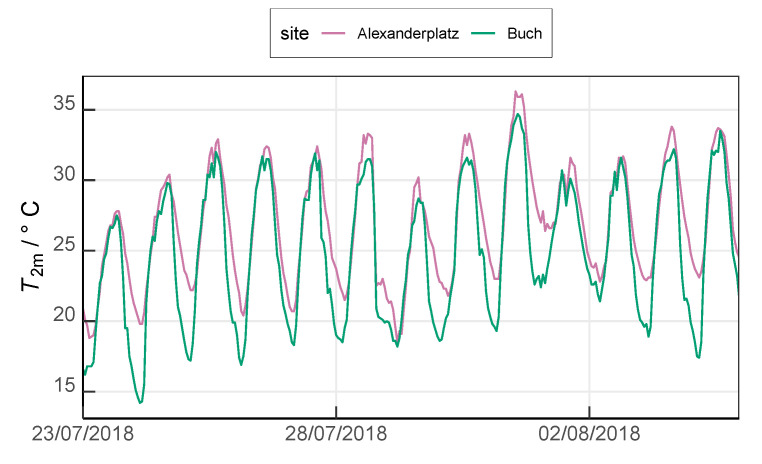
Observed 2 m air temperature at the urban site Alexanderplatz and the suburban site Buch during the analysis period (23 July 2018 0000 UTC to 4 August 2018 2300 UTC). Data stem from measurement sites maintained by the German Meteorological Service (DWD).

During the analysis period (23 July 2018 0000 UTC to 4 August 2018 2300 UTC), a high-pressure system dominated northwestern Europe and led to rather stable atmospheric conditions across Berlin with record-breaking temperatures and drought. The urban site Alexanderplatz experienced higher air temperatures than that at the suburban site Buch (Figure 4). The maximum daily temperature for Alexanderplatz and Buch were recorded as 36.3 ${}^{\circ }C$ and 34.7 ${}^{\circ }C$, respectively. A daily mean temperature of 26.8 ${}^{\circ }C$ was reported at Alexanderplatz and 24.9 ${}^{\circ }C$ at site Berlin-Buch whereas the average temperatures in July from 1981 to 2010 were 20.2 ${}^{\circ }C$ and 19.2 ${}^{\circ }C$, respectively.

## 3. Results

In the following, we present results of the above-mentioned 12 simulations with different AC configurations. We assess the AC scenarios HorAC22 and VerAC22 against the reference scenarios NoAC and AC_noout22 in terms of surface sensible heat flux (Section 3.1), outdoor air temperature (Section 3.2, Section 3.3 and Section 3.4), indoor air temperature (Section 3.5) and energy consumption (Section 3.6). Sensitivity studies using other target indoor temperatures are carried out in Section 3.7.

### 3.1. Sensible Heat Flux at the Surface

The total surface sensible heat flux (QH) is defined as the sum of the heat exchange between the land-and-urban surfaces and the air including the anthropogenic heat from buildings and AC systems. Here, a positive value of QH indicates an energy transport from the surface towards the atmosphere, implying that the temperature of the surface is higher than the air temperature so that the atmosphere is warmed by the surface.

**Figure 5:**
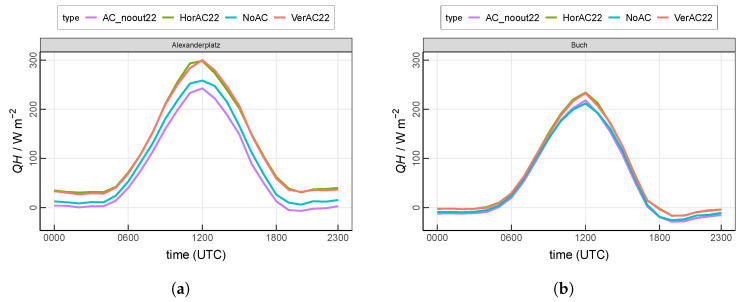
Mean diurnal cycle of the total sensible heat flux including the anthropogenic heat from buildings at (**a**) the urban site Alexanderplatz and (**b**) the suburban site Buch. Values are averaged over the entire 13-day analysis period.

QH shows a similar diurnal cycle at both investigation sites (Figure 5), with its maximum occurring at 1200 UTC and the minimum happening during the night. QH at the urban site Alexanderplatz, which is modeled with the standard reference scenario NoAC, ranges from 6 $W\;m^{-2}$ to 258 $W\;m^{-2}$ (Figure 5a). A considerable increase of QH is simulated in both AC scenarios (HorAC22 and VerAC22) due to the extra waste heat released from the AC into the atmosphere. Compared to the standard reference run NoAC, the maximum increase of QH occurs at noon (by 42 $W\;m^{-2}$ at 1300 UTC). This is caused by the higher energy consumption of AC due to higher insolation and air temperature at noon and the resulting larger amount of waste heat emissions. Despite the different vertical distribution of QH between HorAC22 and VerAC22, the total surface sensible heat flux, however, is similar in both cases. All simulations except AC_noout22 feature a positive QH throughout the night. The scenario AC22_noout has lower QH than NoAC because the indoor temperatures are maintained at 22 ${}^{\circ }C$ and, thus, lower than in the NoAC case, which results in lower urban surface temperatures. Since the waste heat is not considered in AC22_noout, this finding is reasonable but can nonetheless be considered as an artefact.

The suburban site Buch has an overall lower QH (Figure 5b) than the urban site Alexanderplatz due to less impervious surface and more vegetation in the surroundings (cf. Table 2). At night from 1900 UTC to 0400 UTC, negative QH values are observed in all scenarios, indicating an energy transport from the air to the relatively colder surface. QH by NoAC is located in the range of −26 $W\;m^{-2}$ and 212 $W\;m^{-2}$. In contrast to Alexanderplatz, only small differences are depicted among all four scenarios. The largest increase of QH by VerAC22 and HorAC22 occurs at noon by around 22 $W\;m^{-2}$.

### 3.2. AC Contribution to Near-Surface Air Temperature

**Figure 6:**
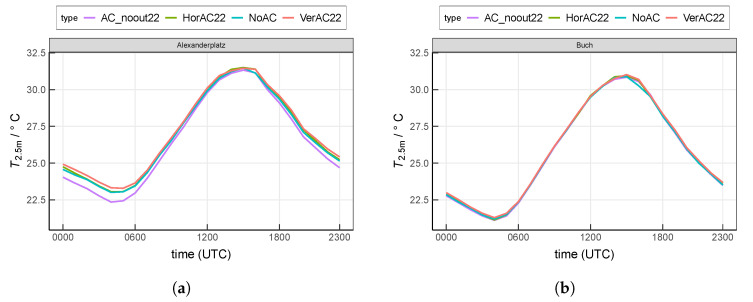
Mean diurnal cycle of near-surface air temperature at the lowest model level at (**a**) the urban site Alexanderplatz and (**b**) the suburban site Buch. Values are averaged over the entire 13-day analysis period.

In the following, the air temperature of the lowest model level ($T_{2.5m}$) is investigated. At site Alexanderplatz, $T_{2.5m}$ in scenario NoAC ranges from 23.0 ${}^{\circ }C$ (0400 UTC) to 31.5 ${}^{\circ }C$ (1500 UTC), as shown in Figure 6a. The scenario VerAC22 produces the highest temperatures especially during the night, which is consistent with QH in Figure 5a. The maximum temperature increase by VerAC22 occurs at 0100 UTC by 0.4 ${}^{\circ }C$ at site Alexanderplatz. The scenario HorAC22 shows a smaller temperature increase (maximum at 0000 UTC by 0.2 ${}^{\circ }C$) compared to the VerAC22 simulation, indicating that VerAC22 has a stronger effect on the air temperature of the lowest model level. In general, AC_noout22 shows the lowest $T_{2.5m}$ among all four scenarios as the low interior temperature of 22 ${}^{\circ }C$ constantly cools the atmosphere. At site Alexanderplatz, $T_{2.5m}$ is on average 0.34 K lower with AC_noout22 than with NoAC.

The suburban site Buch shows generally lower $T_{2.5m}$ than Alexanderplatz, particularly during nighttime (Figure 6b). NoAC produces $T_{2.5m}$ from 21.2 ${}^{\circ }C$ to 30.1 ${}^{\circ }C$. Due to the lower urban fraction at site Buch, compared to the scenario NoAC, the increase of $T_{2.5m}$ simulated with VerAC22 (0.10 K) and HorAC22 (0.05 K) is much smaller than that at site Alexanderplatz. Similarly, $T_{2.5m}$ with AC_noout22 is lower than that with other setups, but the decrease is also very small (−0.03 K compared to NoAC).

### 3.3. Evaluation of AC Contribution within the Boundary Layer

In addition to the the near-surface temperature, we further investigate the air temperatures at different heights and the effect of AC systems on the urban boundary layer.

**Figure 7:**
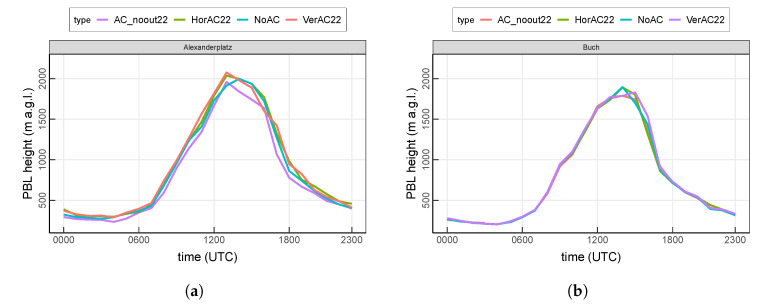
Simulated height of planetary boundary layer (PBL) at (**a**) the urban site Alexanderplatz and (**b**) the suburban site Buch. Values are averaged over the entire 13-day analysis period.

Figure 7 shows the diurnal cycle of the planetary boundary layer (PBL). PBL at Alexanderplatz varies from 234 m–2075 m with a maximum value at 1300 UTC and relatively constant values during the night. The difference among the four scenarios is distinct. Compared to NoAC, the PBL at Alexanderplatz is increased by 36 m and 40 m on average in the HorAC22 and VerAC22 scenarios, respectively. This increase is more pronounced when comparing to AC_noout22, resulting in values of 96 m and 100 m, respectively. PBL at Buch is generally lower than at Alexanderplatz, ranging from 194 m–1896 m. Only a small difference is observed among different scenarios.

**Figure 8:**
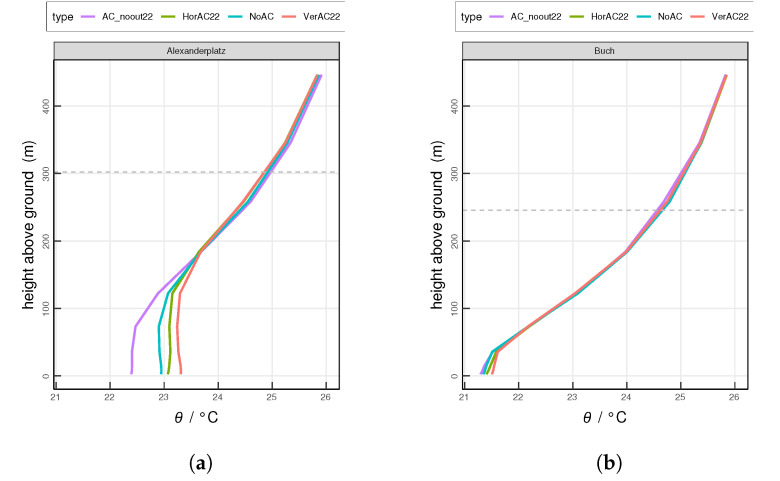
Simulated mean vertical profiles of potential temperature at 0100 UTC at (**a**) the urban site Alexanderplatz and (**b**) the suburban site Buch. Horizontal grey dashed lines indicates the top of the urban height level of the grid cell. The horizontal dashed line indicates the mean PBL height. Values are averaged over the entire 13-day analysis period.

Furthermore, we study the typical nighttime (0100 UTC) and daytime (1300 UTC) vertical profiles of potential temperature θ at both investigation sites. At the urban site Alexanderplatz at 0100 UTC, the values of θ in four scenarios are distinct from the ground surface to around 200 m above the ground (Figure 8a). Among all scenarios, AC_noout22 shows the lowest θ. At the lowest model level, θ by AC_noout22, NoAC, HorAC22 and VerAC22 is 22.4 ${}^{\circ }C$, 22.9 ${}^{\circ }C$, 23.1 ${}^{\circ }C$ and 23.3 ${}^{\circ }C$, respectively. Below 73 m at Alexanderplatz, AC_noout22 shows a slightly increasing θ and NoAC shows a small decreasing trend of θ, indicating that the near-surface atmosphere is lightly stable in scenario AC_noout22 and marginally unstable in NoAC. A slightly unstable atmosphere is observed in HorAC22 up to 35 m. VerAC22 shows a slightly stable one at the same level. From 35 m to 122 m, both HorAC22 and VerAC22 result in a rather neutral atmosphere.

At the suburban site Buch at 0100 UTC (Figure 8b), the difference of θ among different scenarios is less distinctive. θ ranges from 21.3 ${}^{\circ }C$–21.5 ${}^{\circ }C$ at the lowest model level. From the ground to 36 m, θ increases with height in all scenarios, which implies a stable atmosphere. Here, AC_noout22 features the largest stability while VerAC22 shows the least one.

**Figure 9:**
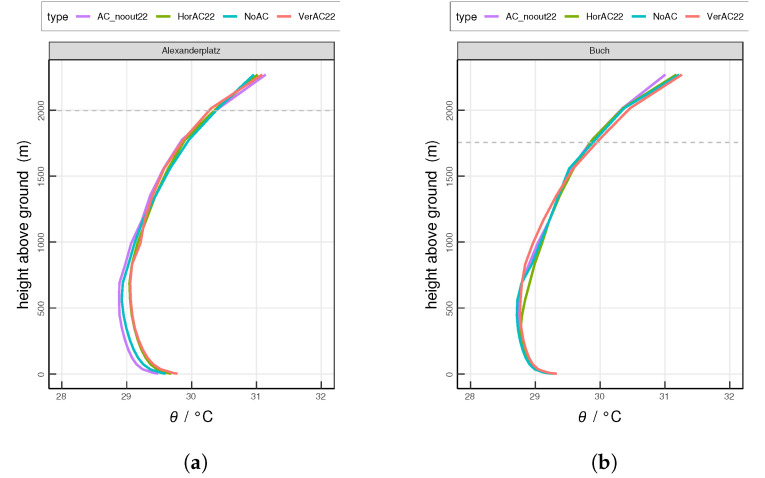
Same as Figure 8, but at 1300 UTC.

The daytime profiles of θ are similar for the two sites (Figure 9). The highest θ occur near the surface and decrease up to 500 m. The near-surface atmosphere is unstable in all scenarios. At Alexanderplatz during the day, while AC_noout22 shows the lowest value of θ within the PBL, the profiles of HorAC22 and VerAC22 do not differ much but show larger values than AC_noout22 and NoAC.

### 3.4. Spatial Distribution of Air Temperature

**Figure 10:**
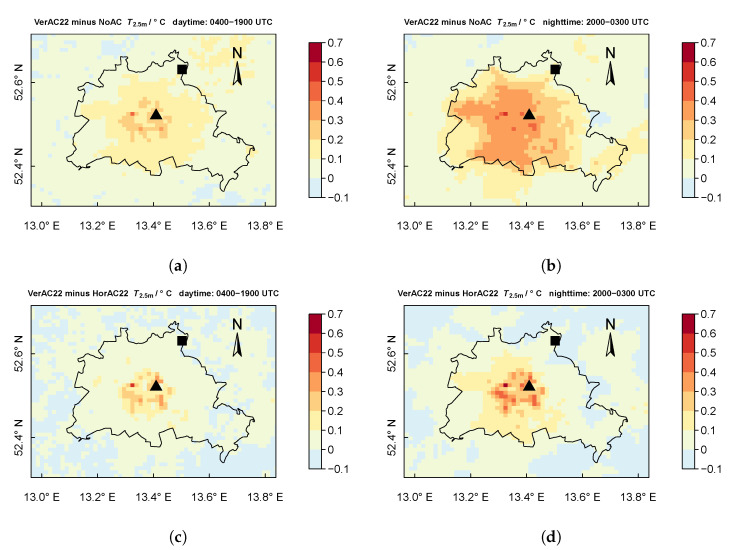
Mean spatial distribution of difference of $T_{2.5m}$ between scenarios. (**a,b**) The temperature difference between VerAC and NoAC during the daytime (0400 UTC to 1900 UTC) and nighttime (2000 UTC to 0300 UTC), respectively. (**c,d**) The temperature difference between VerAC and HorAC. The city border of Berlin is outlined with the black line. The locations of urban site Alexanderplatz (triangle), suburban site Buch (square) are also depicted. The maps use a cylindrical equidistant projection. The grid spacing is about 1 km.

Figure 10a,b compare the scenario VerAC22 with the reference run NoAC regarding $T_{2.5m}$ averaged over daytime (from 0400 to 1900 UTC) and nighttime (from 2000 UTC to 0300 UTC), respectively. We find that $T_{2.5m}$ in the urban areas is increased in VerAC by up to 0.5 K during the day and 0.6 K during the night. Similarly, Figure 10c,d display the difference of $T_{2.5m}$ between two scenarios VerAC22 and HorAC22. The effect of VerAC22 on $T_{2.5m}$ is considerably stronger than HorAC22, notably in the center areas where higher buildings are dominant.

### 3.5. Indoor Temperature with NoAC

**Figure 11:**
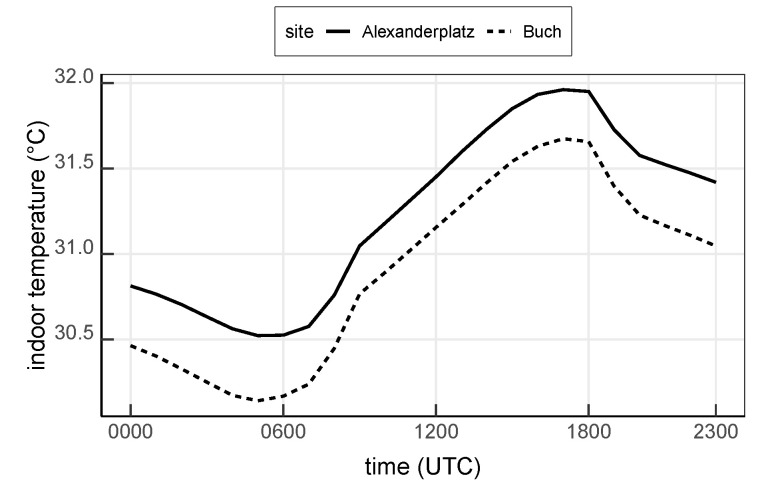
Mean indoor temperature simulated with the reference run NoAC at the urban site Alexanderplatz and the suburban site Buch. Values are averaged over the entire 13-day analysis period.

At site Alexanderplatz, the mean indoor temperature simulated with the reference scenario NoAC ranges from 30.5 ${}^{\circ }C$ to 32.0 ${}^{\circ }C$ with the lowest temperature at around 0600 UTC and the highest at around 1800 UTC. The modeled indoor temperature at site Buch shares a similar diurnal cycle with Alexanderplatz but is about 0.3 K lower. This difference results from the different outdoor air temperature and different building configurations at both sites (cf. Figure 6). In addition, a delayed response of about three hours is observed between the indoor temperature and the outdoor temperature as can be seen from Figure 6 and Figure 11.

### 3.6. Evaluation of AC Energy Consumption

**Figure 12:**
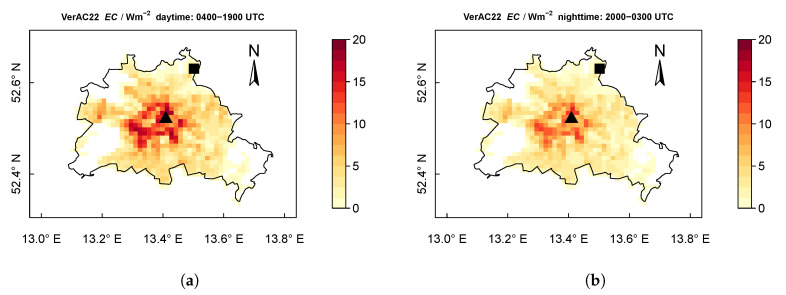

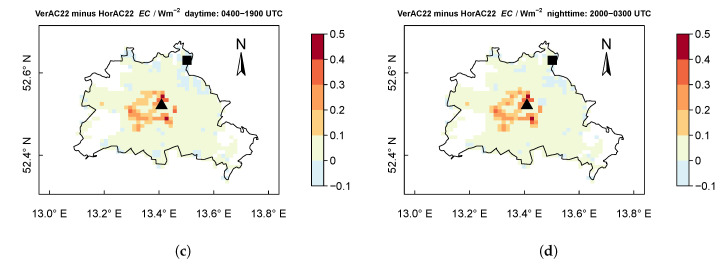
Mean spatial distribution of AC energy consumption (EC) between scenarios. (**a**,**b**) show the mean EC by VerAC22 during daytime (0400 UTC to 1900 UTC) and nighttime (2000 UTC to 0300 UTC), respectively. (**c**,**d**) show the EC difference between VerAC22 and HorAC22 scenarios. The city border of Berlin is outlined with the black line. The locations of urban site Alexanderplatz (triangle) and suburban site Buch (square) are also depicted. Each pixel value represents the EC averaged for the selected hours of the grid cell. The maps use a cylindrical equidistant projection. The grid spacing is about 1 km.

Figure 12 depicts the spatial distribution of the AC energy consumption (EC) of the urban area in Berlin. Each pixel value represents the EC averaged for a grid cell, including the urban part and the vegetated part. During daytime (from 0400 to 1900 UTC), the mean EC in the VerAC22 scenarios is up to 18 $W\;m^{-2}$ at the city center (Figure 12a). For the nighttime hours (from 2000 UTC to 0300 UTC), the maximum mean EC is reduced to 14 $W\;m^{-2}$ (Figure 12b), which is 22% less than that over the day. The averaged EC over the whole area of Berlin during daytime and nighttime is 5.3 $W\;m^{-2}$ and 3.9 $W\;m^{-2}$, respectively. To calculate this spatially-averaged EC, the grid cells with small $f_{urb}$ (<0.1) are excluded, as we mainly focus on areas with considerable urban influence. Figure 12c,d show the EC difference between the scenarios VerAC22 and HorAC22. Compared to HorAC22, VerAC22 shows a slight increase in EC particularly in the central area (up to 0.5 $W\;m^{-2}$).

The main influencing factor of the grid cell average EC is the urban fraction. In order to make the energy consumption more comparable between grid cells of different urban fractions, we consider in the following only the urban part of a grid cell and calculate the AC energy consumption per floor area of a room $EC_{\mathrm{floor}}$ (unit $W\;m^{-2}$) by dividing the total AC energy consumption of an entire building $EC_{\mathrm{total}}$ by the building plan area and the number of the floors of the building:

(1) $$EC_{\mathrm{floor}}=\frac{EC_{\mathrm{total}}}{B\;\cdot \;D\;\cdot \;N_{\mathrm{floor}}}=\frac{EC_{\mathrm{total}}/D}{B\;\cdot \;N_{\mathrm{floor}}}$$

with $EC_{\mathrm{total}}$ (unit W) being the total energy consumption of a building, *B* being the building width, *D* being the canyon length and $N_{\mathrm{floor}}$ being the number of the floors of a building. Note that $E_{\mathrm{total}}/D$ is the output quantity of DCEP-BEM because of the limit D→∞ applied in the model [36].

**Figure 13:**
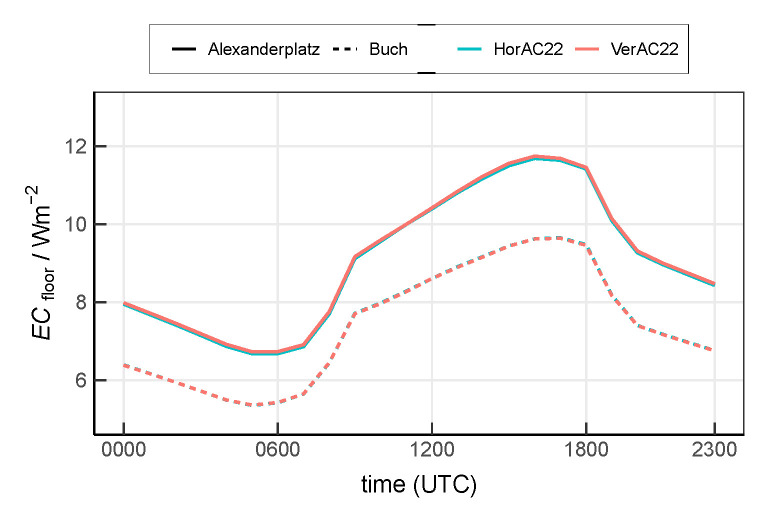
Mean diurnal cycle of $EC_{\mathrm{floor}}$ at the urban site Alexanderplatz and the suburban site Buch by scenario HorAC22 and VerAC22.

Figure 13 shows the mean diurnal cycle. At the urban site Alexanderplatz, $EC_{\mathrm{floor}}$ ranges from 6.7 $W\;m^{-2}$ to 11.7 $W\;m^{-2}$ with the minimum occurring at 0500 UTC and the maximum occurring at 1700 UTC, hours later than the minimum and maximum of the air temperature (Figure 6), as a result of a delayed response of the indoor temperature to the outdoor temperature due to the thermal inertia of the buildings. Compared to the urban site, $EC_{\mathrm{floor}}$ at the suburban site Buch is reduced by 35% on average. The daily mean $EC_{\mathrm{floor}}$ at site Alexanderplatz and Buch are 9.1 $W\;m^{-2}$ and 7.4 $W\;m^{-2}$, respectively. $EC_{\mathrm{floor}}$ simulated with HorAC22 and VerAC22 are almost identical, as the total energy to be released from the whole building is the same. The small difference between both simulation results from the slight difference of the outdoor air temperature (Figure 6).

### 3.7. Sensitivity to the Target Indoor Temperature

**Figure 14:**
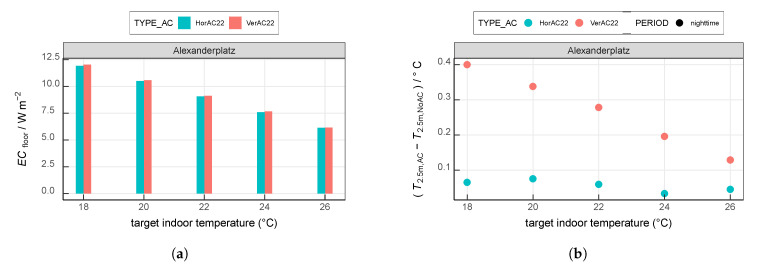
Comparison of simulations with different target indoor temperatures at Alexanderplatz regarding (**a**) mean full-day $EC_{\mathrm{floor}}$ and (**b**) mean difference of nighttime $T_{2.5m}$ between AC and NoAC. Values are averaged over the entire 13-day analysis period.

In this section, we explore the effect of different target indoor temperatures varying from 18 ${}^{\circ }C$ to 26 ${}^{\circ }C$ (Figure 14) at Alexanderplatz. When applying a target indoor temperature of 22 ${}^{\circ }C$, the average full-day $EC_{\mathrm{floor}}$ are 9.1 $W\;m^{-2}$ in the HorAC and VerAC scenarios. When increasing the target indoor temperature to 24 ${}^{\circ }C$, $EC_{\mathrm{floor}}$ is reduced by 16%, simulated with both HorAC and VerAC. When the target indoor temperature is set to 26 ${}^{\circ }C$, 32% $EC_{\mathrm{floor}}$ is saved by both types of AC. When the target indoor temperature is decreased to 20 ${}^{\circ }C$, an additional AC energy consumption of 16% is required compared to the simulations with the indoor temperature of 22 ${}^{\circ }C$. When decreasing the target indoor temperature to 18 ${}^{\circ }C$, we observe an increase in AC energy consumption of 31% and 32% in the HorAC and VerAC scenarios, respectively. The standard deviation of $EC_{\mathrm{floor}}$ for each scenario during the analysis period is 0.8 $W\;m^{-2}$ on average. By applying a paired *t*-test on the daily values, we find a significant difference at significance level of 1% between $EC_{\mathrm{floor}}$ of each VerAC and between each HorAC scenario.

Furthermore, we focus on the effect of different indoor target temperatures on the outdoor near-surface air temperature during nighttime (from 2000 UTC to 0300 UTC) since the daytime temperature is not distinctly changed due to the AC systems (Section 3.2). The nighttime $T_{2.5m}$ simulated with VerAC shows an obvious decrease with increasing target indoor temperatures (Figure 14b). For the target indoor temperature of 18 ${}^{\circ }C$, VerAC computes 0.4 ${}^{\circ }C$ higher $T_{2.5m}$ compared to NoAC. This value decreases with about 0.06 K per 2 K higher target indoor temperature reaching a value of about 0.13 K at a target temperature of 26 ${}^{\circ }C$. The change in nighttime $T_{2.5m}$ simulated with HorAC compared to NoAC is small and fluctuates around 0.05 K. Within the analysis period, the average standard deviation of the temperature increase for the VerAC scenarios and the HorAC scenarios are 0.17 K and 0.10 K, respectively. We find a significant difference at a significance level of 1% between the average temperature increase of each VerAC scenario comparing to any other VerAC scenario using a paired *t*-test on the daily values. The temperature increases of the HorAC scenarios are not significantly different.

## 4. Discussion

**Figure 15:**
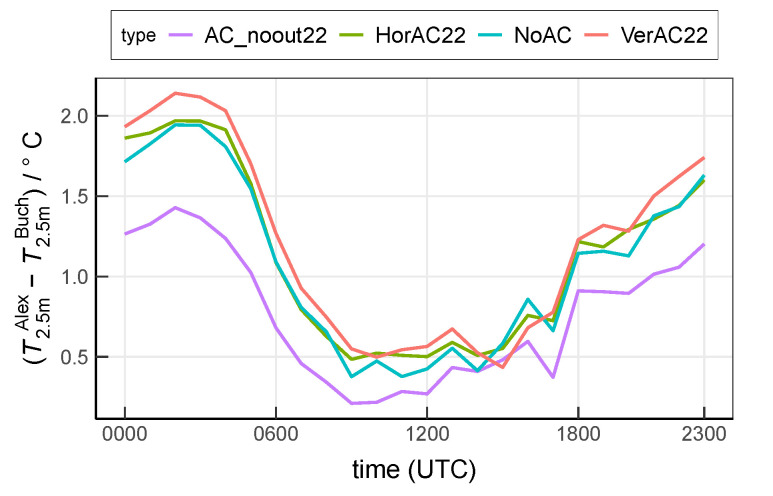
Mean near-surface air temperature difference between Alexanderplatz and Buch. Values are averaged over the entire 13-day analysis period.

Compared to the suburban site Buch, the urban site Alexanderplatz is covered by a larger fraction of impervious surfaces including a larger building fraction, resulting in an overall higher sensible heat flux from surfaces. In the AC enabled scenarios, AC waste heat emissions are added as anthropogenic heat which increases the difference in heat emissions between urban and suburban grid cells even more [26,30]. This leads to an increased difference of near-surface temperature between Alexanderplatz and Buch (Figure 15). While the total, vertically summed-up sensible and anthropogenic heat flux is approximately equal in the VerAC22 and in the HorAC22 scenario, it is distributed vertically differently in the two scenarios. In the case of VerAC22, more heat is emitted to the lower levels so that the temperature of the lowest model level increases more than in the HorAC22 case. Since only 5% of the buildings at Alexanderplatz (Table 2) have their rooftops located within the lowest model level (5 m), the amount of the heat release from the rooftop of the 5 m-buildings is very small. Consequently, the respective temperature difference between Alexanderplatz and Buch is larger in the VerAC22 case than in any other scenario.

The scenario AC_noout represents an idealized case which keeps the indoor temperature at a certain temperature but without waste heat ejected into the atmosphere. With help of AC_noout, the impact of AC waste heat can be evaluated explicitly [20,21,30]. When compared to AC_noout22, the extra AC waste heat increases the air temperature at Alexanderplatz by 0.9 K (VerAC22) and 0.7 K (HorAC22) on average during the night (2000-0300 UTC). Here, it has to be noted that air temperature in the NoAC scenario is higher than in the AC_noout22 scenario especially during nighttime (up to 0.7 K) due the cooler indoor environment. Comparable values are reported in [26,30]. In general, the increase in air temperature due to AC is more pronounced during the night despite that the largest heat flux increase occurs around noon. Martilli et al. [30] argue that the lower PBL height and the relatively larger proportion of the AC waste heat to the total heat emission during the night are reasons for that.

At Alexanderplatz at 0100 UTC, a mixed layer (ML) is formed from the ground up to 100 m in the scenarios AC_noout22 and NoAC (Figure 8a). This weakly-convective nocturnal ML results from the upward sensible heat flux (Figure 5a) by urban structures [64]. The height of the ML as simulated with HorAC22 and VerAC22 is increased to around 150 m due to the extra AC waste heat emissions. The atmospheric instability increases the thermal turbulence and promotes air mixing. Compared to HorAC22, VerAC22 shows a more unstable atmosphere near the ground at site Alexanderplatz, implying that the air pollutants emitted near the surface may be spread vertically faster in the VerAC22 scenario. At the suburban site Buch at night, the ML is absent, as the mean nighttime sensible heat flux is not upward, but from the atmosphere towards the surface (Figure 8b). Instead, a nocturnal stable layer (NSL) is modeled from the ground to around 50 m. The atmosphere within the NSL simulated with AC_noout22 is most stable while with VerAC22 is almost neutral. In summary, the AC waste heat at night weakens the stability of the atmosphere. During daytime at 1300 UTC, both sites show typical daytime profiles with a pronounced ML (Figure 9). Stronger mixing processes occur over Alexanderplatz compared to Buch due to stronger AC waste heat release in the city.

**Figure 16:**
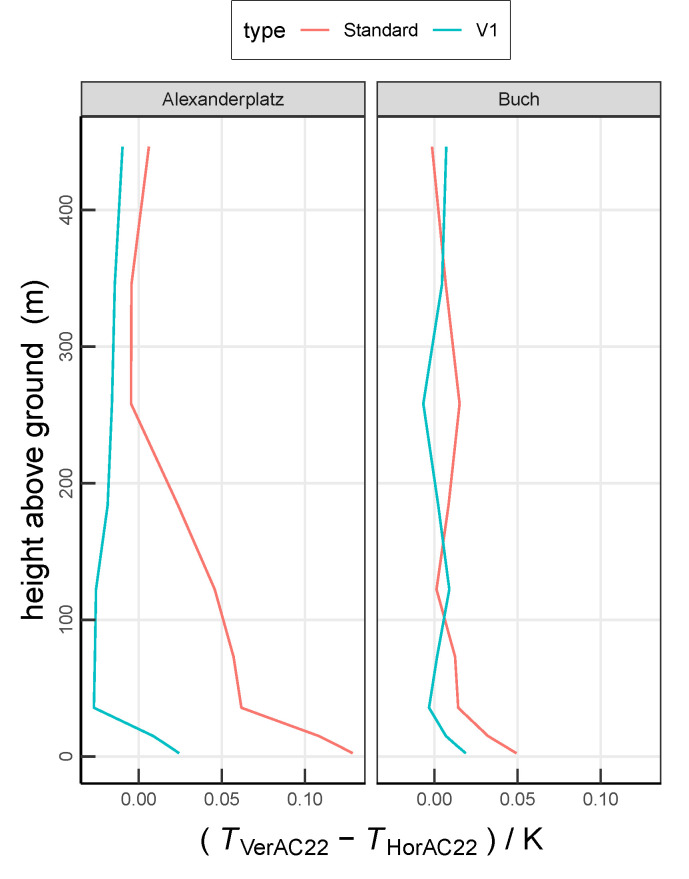
Vertical mean temperature difference between VerAC22 and HorAC22; “Standard” refers to the simulation carried out in this study. “V1” indicates the test simulations with the full atmospheric layers without removing the volume of the buildings. Values are averaged over the entire 13-day analysis period.

A limitation of this study becomes apparent from Figure 8 and Figure 9. While VerAC22 predicts a warmer air temperature than HorAC22 throughout the PBL, we expect warmer air temperatures in the HorAC22 scenario at the heights where the roofs are dominant. This inconsistency is likely due to different vertical transport and advection within both simulations. In the DCEP-BEM scheme, the volume of air within a model level is reduced by the volume of the buildings at each level [51]. The reduction in air volume due to the building volume at that level increases the effect of the sensible heat flux on the air temperature at that specific level. Since the fraction of buildings is larger in lower model levels than in higher model levels (cf. Table 2), the effect of the volume reduction is more pronounced in lower model levels. However, CCLM does not consider a reduction of volume through buildings when advection and vertical diffusion are computed. This leads to overestimated vertical transport due to an overestimation of the involved air volume, which consequently probably leads to overestimated the air temperature at the rooftop level in the VerAC22 scenario compared to the ground level. In order to investigate this phenomenon, we carry out two test simulations of HorAC22 and VerAC22 considering the full atmospheric layers without removing the volume of buildings in DCEP-BEM scheme (V1 in the following). This removes the overestimation of the effect of the vertical transport but does underestimate the temperature change due to sensible heat flux at each level. Figure 16 compares the difference between VerAC22 and HorAC22 for this new simulation and the default setting. As already noted, at Alexanderplatz, we find that standard HorAC22 shows lower temperatures than standard VerAC22 around the level of 36 m where the majority of rooftops of buildings of this grid cell is located which actually is an artefact. In contrast to this, in the V1 simulations, and as expected, HorAC22 is warmer than VerAC22 above 36 m. However, the near-surface temperatures of V1 simulations are too low; NoAC_V1 is about 0.3 ${}^{\circ }C$ cooler during nighttime due to the larger air volume in the DCEP-BEM scheme than standard NoAC which leads to an underestimation of the urban heat island intensity by NoAC_V1 (cf. the detailed evaluation of NoAC in Jin et al. [36]).

For the area of Berlin, the modeled AC energy consumption (EC) during the daytime hours amounts up to 4.6 $W\;m^{-2}$, which is 39% more than the mean EC during the night. In the central areas where the urban surface and higher buildings are dominant, higher EC values are observed than the surroundings (Figure 12).

**Figure 17:**
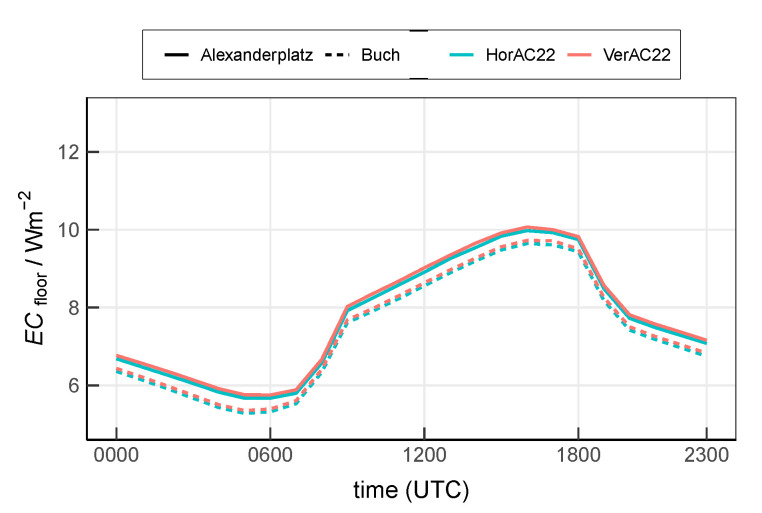
Mean diurnal cycle of $EC_{\mathrm{floor}}$ at the urban site Alexanderplatz and the suburban site Buch with the same building parameters for all grid cells.

In this study, the AC consumption shows a clear diurnal cycle with the minimum after the morning transition and the maximum in the late afternoon (Figure 13). Similar findings have been reported in Salamanca et al. [20] and Salamanca et al. [21]. The mean AC consumption (Figure 13) amounts to 7.5 $W\;m^{-2}$ and 9 $W\;m^{-2}$ per floor area of room ($EC_{\mathrm{floor}}$) for Alexanderplatz and Buch, respectively, implying a positive difference of 19% at Alexanderplatz compared to Buch. This increase is partly due to the UHI but also due to the different building parameters, in particular the building and street widths. Therefore, in order to study the influence of the setting of the sites only, we conduct additional simulations in which the building widths and the street widths of all grid cells are set to 17 m and 25 m, respectively, and all four street directions are evenly distributed. In these simulations (Figure 17), $EC_{\mathrm{floor}}$ behaves qualitatively similarly to our previous findings (Figure 13). The mean $EC_{\mathrm{floor}}$ is about 7.8 $W\;m^{-2}$ and 7.5 $W\;m^{-2}$ at Alexanderplatz and Buch, respectively, in the VerAC22 case and each about 0.1 $W\;m^{-2}$ lower in the HorAC22 than in the VerAC22 cases. Thus, we find a difference of about 4% when comparing urban and suburban setting only.

Various studies have estimated the AC energy consumption and analyzed the increase of the cooling load caused by UHI. [20,21] report the AC consumption for Phoenix of up to 3 $W\;m^{-2}$. This value is less than in our study because the buildings in Phoenix are 1 to 2 floors and are with very low density. The UHI impact on building energy performance has a broad spectrum based on previous studies. Santamouris [65] derived an average increase of 13% due to UHI in general. Cui et al. [66] concluded a similar rate of 11% for Beijing. Based on Xu et al. [23], an increase of 31% and 20% is observed in Beijing for an urban office building and an urban residential building, respectively, from suburban simulations. Concerning residential buildings, the sensible cooling load is increased by UHI intensity by 18% to 28% in Barcelona [19]. Compared to the rural case, 41% of the cooling load is increased for a non-insulated building in Milan [18]. All of these values fall in the same order of magnitude and are comparable with the results for Berlin reported here.

## 5. Conclusions

This study investigates the effect of AC systems on the urban environment in Berlin (Germany) during a summer period using the urbanized regional climate model CCLM/DCEP-BEM. We consider two different configurations of the AC systems: with vertical AC outdoor units (VerAC), the waste heat emission of each floor is released into the outdoor air of corresponding height. Horizontal AC outdoor units (HorAC) release the total waste heat of a whole building at the height of its rooftop.

The AC systems considerably increase the total surface sensible heat flux (QH) by ejecting waste heat into the atmosphere. For the scenarios with the target indoor temperature of 22 K, compared to the reference simulation without AC, both VerAC and HorAC increases QH by about 30 $W\;m^{-2}$ at the urban site Alexanderplatz and around 10 $W\;m^{-2}$ at the suburban site Buch. The waste heat generated by VerAC and HorAC are very similar, as the amount of the energy to be extracted is the same but only vertically distributed in different ways.

The effect of VerAC and HorAC on air temperatures are explored temporally and spatially. During nighttime, the increase of air temperature due to AC is more pronounced. In the urban area of Berlin, the near-surface air temperature is increased by up to 0.6 K during the nighttime and up to 0.5 K during the daytime in the VerAC scenario and a target indoor temperature of 22 ${}^{\circ }C$. On the vertical scale, VerAC produces a noticeable increase in air temperature. At Alexanderplatz, the effect of HorAC is overall smaller than VerAC and increases from the bottom level until the maximum building height. VerAC increases the atmospheric instability near the ground and consequently promotes the vertical mixing of air pollutants.

The maximum values of AC energy consumption with about 18 $W\;m^{-2}$ are observed in the central areas of Berlin with denser and higher buildings. While during the day, the mean AC energy consumption for Berlin is 4.6 $W\;m^{-2}$ on average, during nighttime about 35% of the energy is saved. With the target indoor temperature of 22 ${}^{\circ }C$, the AC energy consumption per floor area of a room at the urban site Alexanderplatz ranges from 6.7 $W\;m^{-2}$ to 11.7 $W\;m^{-2}$, 23% more than at the suburban site Buch. This difference results from the urban heat island as well as different building parameters at the two sites. With equal building parameters, we find a difference of 4% between the urban and suburban site.

Both the AC energy consumption and the near-surface temperature increase depend on the target indoor temperature. In the range of 18 ${}^{\circ }C$ to 26 ${}^{\circ }C$, the AC energy consumption decreases by about 15% per 2 K target indoor temperature, and is significantly different between scenarios. The nighttime near-surface temperature increases in the VerAC scenario by about −0.05 K per 2 K. A significant difference at a 1% significance level is shown between VerAC scenarios on the temperature increase. The change of the near-surface temperature in the HorAC scenario is small, indicating that the HorAC has little impact on the near-surface temperature. The temperature increase in the HorAC scenarios are not significantly different.

This study presents an attempt to compare the effects of different AC cooling systems on urban climate in a modeling approach that could be beneficial for urban planning in terms of energy savings and UHI mitigation. Each additional degree of AC cooling raises the UHI effects and increases the energy demand, which adversely affects both outdoor climate adaptation and energy savings. Moreover, for the future urban planning, in order to mitigate the increasing UHI, horizontal AC is preferred over vertical AC since the latter have a large impact on near-surface air temperature and UHI while this impact is much smaller for horizontal AC. It is worth noting that when applying horizontal AC on rooftops, with the same building volume, it is better to have few very tall buildings instead of setting-up densely quarters with low-rise buildings of only some floors as it is unfortunately preferred in contemporary building construction activities in Berlin. Future studies could be addressed in more realistic applications and scenarios with more specific types of AC, and with a link to related research questions in the field of air quality.

## Figures and Tables

**Table 1:** Physical parameterizations of COSMO-CLM (CCLM) simulations.

Time integration	Wicker and Skamarock [40]
Planetary boundary layer scheme	Mellor and Yamada [41] and Raschendorfer et al. [42]
Lateral boundary conditions	Davies [43]
Radiation scheme	Ritter and Geleyn [44]
Convection	Tiedtke [45] scheme for CCLM-7 km; a shallow convection scheme for CCLM-1 km
Microphysics scheme	Kessler [46]
Spectral nudging	Rockel et al. [47]

**Table 2:** Parameters of selected urban model grid cells. $f_{urb}$: urban fraction. *W*: average street width (m). *B*: average building width (m). $\gamma_{i}$: building height distribution.

				$\gamma_{i}$									
Site	$f_{urb}$	W/m	B/m	0 m	5 m	10 m	15 m	20 m	25 m	30 m	35 m	40 m	45 m
Alexanderplatz	0.69	29.80	14.70	0.00	0.03	0.02	0.04	0.19	0.41	0.26	0.02	0.00	0.04
Buch	0.39	35.30	19.50	0.01	0.12	0.25	0.32	0.23	0.00	0.02	0.05	0.00	0.00
